# Unveiling Weyl-related optical responses in semiconducting tellurium by mid-infrared circular photogalvanic effect

**DOI:** 10.1038/s41467-022-33190-3

**Published:** 2022-09-15

**Authors:** Junchao Ma, Bin Cheng, Lin Li, Zipu Fan, Haimen Mu, Jiawei Lai, Xiaoming Song, Dehong Yang, Jinluo Cheng, Zhengfei Wang, Changgan Zeng, Dong Sun

**Affiliations:** 1 International Center for Quantum Materials, School of Physics, Peking University, Beijing, 100871 P. R. China; 2 International Center for Quantum Design of Functional Materials, Hefei National Laboratory for Physical Sciences at the Microscale, University of Science and Technology of China, Hefei, Anhui 230026 P. R. China; 3 Synergetic Innovation Center of Quantum Information & Quantum Physics, University of Science and Technology of China, Hefei, Anhui 230026 P. R. China; 4 Chinese Academy of Sciences Key Laboratory of Strongly Coupled Quantum Matter Physics, Department of Physics, University of Science and Technology of China, Hefei, Anhui 230026 P. R. China; 5 State Key Laboratory of Precision Measurement Technology and Instruments, School of Precision Instruments and Opto-electronics Engineering, Tianjin University, Tianjin, 300072 P. R. China; 6 Changchun Institute of Optics, Fine Mechanics and Physics, Chinese Academy of Sciences, Changchun, 130033 China; 7 Collaborative Innovation Center of Quantum Matter, Beijing, 100871 China

**Keywords:** Topological matter, Topological matter, Nonlinear optics

## Abstract

Elemental tellurium, conventionally recognized as a narrow bandgap semiconductor, has recently aroused research interests for exploiting Weyl physics. Chirality is a unique feature of Weyl cones and can support helicity-dependent photocurrent generation, known as circular photogalvanic effect. Here, we report circular photogalvanic effect with opposite signs at two different mid-infrared wavelengths which provides evidence of Weyl-related optical responses. These two different wavelengths correspond to two critical transitions relating to the bands of different Weyl cones and the sign of circular photogalvanic effect is determined by the chirality selection rules within certain Weyl cone and between two different Weyl cones. Further experimental evidences confirm the observed response is an intrinsic second-order process. With flexibly tunable bandgap and Fermi level, tellurium is established as an ideal semiconducting material to manipulate and explore chirality-related Weyl physics in both conduction and valence bands. These results are also directly applicable to helicity-sensitive optoelectronics devices.

## Abstract (web summary)

Materials with Weyl cones and band gaps coexisting are desired due to their combination of Weyl cones and the tunable and controllable nature of semiconductor. Here, the authors find Weyl-related band structures and semiconducting gap of tellurium.

## Introduction

Weyl semimetals have attracted tremendous research interests due to their exotic topological properties^1–7^. These novel topological features, which arise from their nontrivial band structures, have exhibited great potential for high-performance electronic and optoelectronic devices. Compared to (semi)metals, semiconductors and atomic thin layered materials are established material platforms for functional devices, highlighting versatile tunable properties through convenient control approaches, such as doping and electric gating. Previously, Weyl-cone-related topological properties have been generally regarded as an exclusive nature of semimetallic materials, with the nodal points located around the Fermi level. A material platform combining the favorable features of semiconductor and Weyl properties is of great significance for both fundamental physics studies and functional topological devices.

Recently, tellurium (Te), a narrow bandgap semiconductor (~0.28~0.38 eV)^8–10^, has been rediscovered as a candidate of so called “Weyl semiconductor”^11^, where semiconducting energy gap and Weyl cones coexist, with Fermi level lying at the vicinity of both band edge and Weyl cones. Combining advantages of exotic Weyl physics and versatile semiconducting properties, Te has aroused enormous research interests in the field^11,12^. Strong spin-orbit interaction of Te leads to complex spin texture and spin-splitting electronic bands. Crossings of these nondegenerate bands form multiple Weyl cones in both the conduction and valence bands^9,12–15^.

In previous studies, self-hole-doping of bulk Te crystals makes the Fermi level lying at the vicinity of Weyl cones near the valence band edge, facilitating the observation of Weyl-related intriguing magneto-transport behaviors, such as chiral-anomaly-induced negative longitudinal magnetoresistance (NLMR) and the planar Hall effect (PHE)^11^. In addition, the topological response of Weyl cone in the conduction band can also emerge when the Fermi level is tuned across the energy gap in Te flakes^12^. For these transport measurements, many trivial effects need to be carefully ruled out to give a smoking gun evidence of the topological origin of transport signatures^16–19^. Spin-resolved ARPES, a powerful tool to reveal the spin character of electronic bands, have been applied to study the unconventional spin texture of Weyl cones in Te^20^. However, the helicity-dependent optical selection rules of optical transitions, which is critical to the Weyl physics-related optical response, cannot be completely determined by spin texture alone due to the significant contribution of orbital angular momentum in Te, thus it remains to be elusive experimentally.

Alternatively, the helicity-resolved photocurrent measurement is suitable to characterize chirality-related features of optical responses. It is sensitive to the chirality of individual Weyl cone and relies little on the doping condition, therefore it acts as a flexible and direct probe to explore optical properties and Weyl physics previously^6^. In addition, photocurrent responses can be directly utilized in optoelectronics devices, especially photodetectors, a device perspective that Weyl physics locates its promising applications^21^. Attractively, the coexistence of a bandgap and multiple Weyl crossings in conduction/valence bands of Te can lead to abundant helicity-dependent optical selection rules within and between Weyl cones. These helicity-related selection rules can be revealed by an experimentally measurable circular photo galvanic effect (CPGE)^5^. CPGE arises from a transfer of the angular momentum from photons to electrons leading to an oriented photocurrent. It has been observed in systems with unique spin textures and low symmetry such as zinc-blende quantum wells^22,23^. CPGE also serves as an established experimental approach to study topological insulators^24,25^ and Weyl semimetals^6,26–36^. Strong spin orbit interaction and low crystal symmetry make Te an ideal platform for CPGE studies back to 1970s^37–39^, and the CPGE observed in these early experiments are attributed to intraband transitions within valence bands after 10.6 μm photons were absorbed by free carriers. However, these early CPGE studies are quite limited when the topological nature of Te was not aware. In this work, we perform circular-polarization-dependent photocurrent measurements at several different wavelengths in mid-infrared range to probe the chiral optical selection rules of the transitions between Weyl bands in Te. Although the lattice structure of Te has fixed helicity^9,40^, which usually determines the sign of helicity-dependent response such as optical activity et al.^14^, the sign of CPGE response is found to be wavelength dependent in our experiment, as dominated by helicity-dependent optical selection rules. Two typical optical transitions: one transition within a single Weyl cone in the valence band, and the other transition between two Weyl cones in valence and conduction bands, can happen when excited with different wavelengths. These two transitions can only be excited by light of opposite helicities, which leads to sign switch of CPGE that cannot be explained by the helical lattice structure of Te. This is the first time CPGE is firmly detected from transitions between multiple Weyl cones across an energy gap. We further verify the response is a second-order nonlinear effect which rules out the possibility that the sign switch is a trivial effect due to the sign switch of the built-in electric field through a third-order nonlinear effect as reported in the previous work^27^. Furthermore, we show this effect is highly tunable with a back-gate, showing flexible tunability comparing to semimetals, thanks to the existence of a semiconducting band gap in Te.

## Results

### Crystal and electronic band structures of Te

**Fig. 1:**
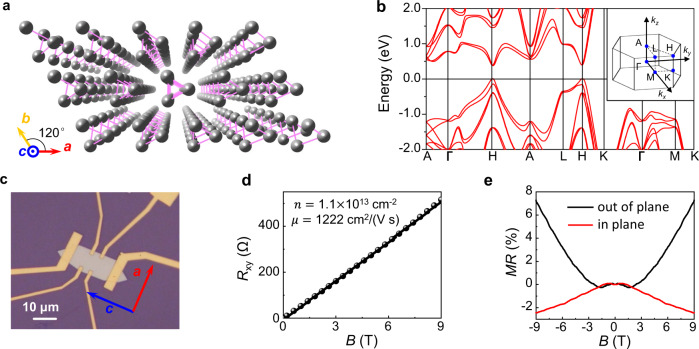
Basic characterization of Te flakes and experimental configuration of photocurrent measurement. **a** The lattice structure of Te. **b** The energy dispersion of electronic bands in Te. Inset shows the corresponding Brillouin zone. **c** The optical microscopy of a Hall-shaped Te device. Red and blue arrows denote to crystallographic *a* and *c* axes, respectively. **d** Hall and **e** magnetoresistance measurements of Te devices.

**Fig. 2:**
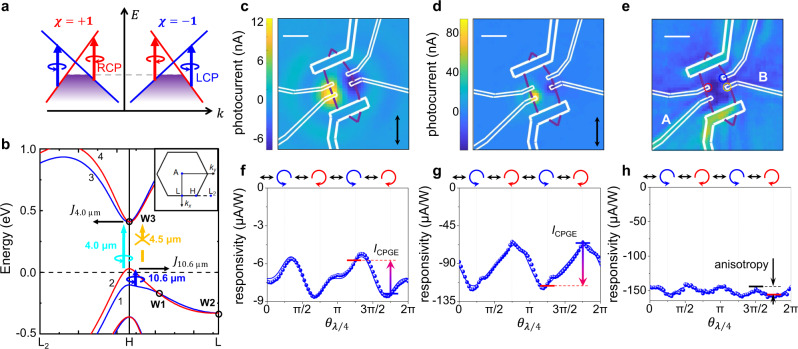
Contrasting circular-polarization-dependent photoresponse of Te under different mid-infrared wavelength excitations. **a** A schematic diagram of optical selection rules at the vicinity of a pair of Weyl nodes with opposite chirality. The gray dash line marks the Fermi level. **b** The band diagram near the H point of Te taking spin-orbit interaction into consideration. The blue, cyan, and yellow arrows denote to transitions induced by 10.6 μm, 4.0-μm and 4.5-μm excitations, respectively. W1, W2, and W3 marks three Weyl nodes near H point. Energy bands forming W1 and W3 are marked by 1–4. Inset shows Brillouin zone with L, H, and L_2_ points. **c**, **d** Scanning photocurrent images of the Te device under 10.6-μm and 4.0-μm excitation, respectively. Double-arrows denote to the direction of excitation light polarization. **e** The scanning reflection image of the Te device under 4.0-μm excitation. The yellow, red, and blue rings mark positions where ${\theta }_{\lambda /4}$-dependent photocurrent is measured. All the scale bars in (**c**–**e**) are 10 μm. **f**, **g** ${\theta }_{\lambda /4}$-dependent photocurrent responsivity under 10.6-μm and 4.0-μm excitation, respectively. Arrows on top of the panels illustrate the polarization sequences, where blue and red circles represent left and right circularly polarizations, respectively. Vertical arrows represent the difference in photocurrent between RCP and LCP excitation, which determines the amplitude of t**h**e CPGE component. **h** ${\theta }_{\lambda /4}$-dependent photocurrent responsivity under 4.5-μm excitation.

Te crystal possesses a trigonal crystal lattice, composed of three-fold helical atomic chains (Fig. 1a). These chains are parallel to the crystallographic *c*-axis. With right- or left-handed helical chains, the lattice structures of Te belong to ${D}_{3}^{4}$ or ${D}_{3}^{6}$ point groups, respectively^9,40^. Inversion and mirror symmetries are both absent in this material, making it an ideal platform for studies on chirality-related properties. Past work has already confirmed that Te is a narrow bandgap semiconductor. An indirect bandgap ranging from 0.28 to 0.38 eV has been reported in the literature depending on the specific synthesis method^8,10,11,40–42^. Strong spin-orbit coupling is hosted in Te, so that the spin degeneracy of both the lowest conduction band and the uppermost valence band is lifted^40^. As shown in Fig.1b, the semiconducting bandgap appears at H point (inset) and Weyl cones exist along the H-L direction (inset). More detailed band structures near the bandgap edge and the band crossings forming Weyl cones are shown in Fig. 2b.

### Characterization of Te devices by transport measurement

Te flakes used in this work were grown via a solution-phase synthesis process (as described in the method session). The long edge of the as-grown samples is along the crystallographic *c* axis. A standard Hall bar device was then fabricated, with the source and drain electrodes along the long edge (Fig. 1c). Figure 1d, e show the typical transport characterizations performed at 1.5 K. The flake is slightly hole-doped with a carrier density of 1.1$\times$10^13^ cm^−2^, which corresponds to the Fermi level lies ~40 meV below the highest valence band maximum, and the carrier mobility is further determined to be above 1200 cm^2^/(V s). Besides, differing from the conventional positive magnetoresistance (MR) behavior when the magnetic field is perpendicular to the current, negative MR is observed under the parallel magnetic field (Fig. 1e), which may be induced by the chiral anomaly effect according to the previous work^3,11,40,43^(more transport results presented in Supplementary Fig. 1).

### Chirality-related optical selection rules in Te

The optical response of Weyl cones is determined by the chirality-related optical selection rules during interband optical transitions, which essentially originate from the conservation of the total angular momentum of photon and electron^5^. For a typical Weyl cone in Weyl semimetals deriving from a two-band Hamiltonian, transitions from spin-up to spin-down bands require a left circular polarized (LCP) excitation, while those from spin-down to spin-up bands require a right circular polarized (RCP) excitation. Therefore, photons with different helicities can only be absorbed at opposite sides of one Weyl cone in a spin-flip transition as determined by the chirality (Fig. 2a). Circular-polarization-dependent photocurrent can then be generated from a single Weyl cone. However, Weyl cones usually appear in pairs in a Weyl semimetal so that photocurrent generated from a pair of Weyl cones should vanish (Fig. 2a). In materials with reduced symmetries, asymmetric transitions happen near Weyl cones due to the tilting of energy bands and Pauli blockade^5,6^. As a result, photocurrent generated from a pair of Weyl cones cannot cancel each other and nonvanishing CPGE exists. The direction of photocurrent switches when the helicity of the incident light switches between LCP and RCP. Consequently, CPGE can act as a sensitive probe for optical transitions relevant to chirality-determined selection rules^6^.

In Te, two sets of Weyl cones formed in valence and conduction bands together with slight self-hole-doping provide a more complicated while more interesting platform for CPGE study. As shown in the detailed band diagram (Fig. 2b), the strong spin-orbit coupling (SOC) leads to spin-splitting energy bands as shown by the red and blue lines, respectively. Weyl cones W1 and W2 are formed by crossings of spin-splitting valence bands and W3 is formed by crossing of spin-splitting conduction bands^40^. As a characteristic property, a hedgehog spin texture is formed near W3 in the conduction bands^9,12,44^. Different from Rashba spin-splitting in quantum wells^22,23^ and bulk states of topological insulators^25^, directions of spins in conduction bands of Te are oriented radially. Due to strong SOC, the spin angular momentum is no longer a good quantum number in Te. Considering that electrons in Te hold a much larger orbital angular momentum compared to the spin one^14^, the orbital angular momentum may also play a role in determining helicity-dependent optical selection rules. Therefore, the optical selection rules of transitions in Te go beyond a two-band Hamiltonian and the general chirality optical selection rules near a Weyl cone described in Fig. 2a no longer applies to Te. Alternatively, we perform numerical calculations to determine the chirality selection rules of optical transitions within or between Weyl cones to include contributions from both spin and orbital angular momentums. We numerically calculate the optical matrix elements as well as tensor elements for injection current (corresponding to CPGE), as described in Supplementary Section 2. Both approaches provide consistent results regarding that helicity-sensitive photocurrent can be generated and collected along opposite directions during two different optical transitions. The first kind of transitions can happen within Weyl cone W1 in the valence band. This transition is from energy band 1 to 2 (1$\to$2) at one side of W1 cone along the H-L direction as shown by the blue arrow in Fig. 2b. LCP light is preferable to induce such an interband transition according to chirality selection rules. Transitions from the other side of W1 are forbidden as both bands are buried deeply under the Fermi level. The same situation applies to any transitions within W2 and W3 cones. The second kind of helicity-sensitive transition happens between W1 and W3 cones across the bandgap. The transition is from energy band 2 to 3 (2$\to$3) as shown by the cyan arrow in Fig. 2b. LCP light is preferable for this transition along the H-L_2_ direction according to chirality selection rules, so 2$\to$3 also provides spin orientation-induced CPGE response. Experimentally, we will focus on transitions 1$\to$2 and 2$\to$3. These two transitions occur with different photon energies. The LCP light always leads to a higher probability of transitions and the generated photocurrent is along the opposite direction, so that the sign of CPGE generated during these two transitions is opposite. This feature is crucial to distinguish these two categories of transitions, both of which can be excited in mid-infrared wavelength range.

### CPGE response under 10.6-μm and 4.0-μm excitations

The 1$\to$2 and 2$\to$3 transitions and the CPGE responses related to them are studied at 10.6 and 4.0 μm respectively. Firstly, scanning photocurrent measurements are carried out to determine regions where the photoresponse can be generated. Similar distributions of photoresponse are observed under both 10.6-μm and 4.0-μm excitations as shown in Fig. 2c, d, when compared with simultaneously recorded scanning reflection images as shown in Fig. 2e. Photocurrents of opposite signs occur near the two electrodes A and B. Then the circular-polarization-dependent photoresponse measurement is carried out by continuously rotating a quarter wave-plate (QWPL) and the photocurrent is recorded as a function of rotation angle of QWPL (${\theta }_{\lambda /4}$). Firstly, lasers are focused near the electrode B as shown by the green circle in Fig. 2e, so that the peak of negative photoresponse along crystallographic *a*-axis is recorded. Polarization-dependent photocurrents under 10.6-μm and 4.0-μm excitations are shown in Fig. 2f, g, respectively. The magnitude of photocurrent is different under LCP and RCP excitations, and shows a clear 180-degree period with respect to ${\theta }_{\lambda /4}$ (see Fourier transform in Supplementary Fig. 4). These features indicate the existence of CPGE. For 10.6-μm excitation, the photoresponse is stronger under RCP excitation compared to that under LCP excitation, while for 4.0-μm excitation, the photoresponse is stronger under LCP excitation compared to that under RCP excitation, which is exactly opposite to that of 10.6-μm excitation. We further note the ${\theta }_{\lambda /4}$-dependent photocurrent response has a 90-degree period component, which corresponds to an anisotropic response between crystallographic *a-* and *c*-axes (see Fourier transform in Supplementary Fig. 4). The contrasting feature, namely opposite directions of CPGE under 10.6-μm and 4.0-μm excitations, is fully consistent with the chiral selection rules described in the previous paragraph. These phenomena are also consistent with numerical calculations (see calculated optical matrix elements and tensor elements for injection current in Supplementary Fig. 2). Thus the CPGE response demonstrated in this work provides a clear evidence about chirality-related optical selection rules for transitions between Weyl cones in both valence and conduction bands (results of other devices presented in Supplementary Figs. 5–8).

As a further verification measurement, the excitation photon energy is tuned down from 310 meV (4.0 μm) to 276 meV (4.5 μm), which is slightly below the lower bound of energy gap. The ${\theta }_{\lambda /4}$-dependent measurement of 4.5-μm excitation shows no CPGE response (Fig. 2h), although a nonzero photoresponse is still detected. The photoresponse may originate from transitions in the valence band which are irrelevant to the helicity-dependent selection rules. On the other hand, the thermoelectric effect may also contribute to the photocurrent response at 4.5 μm. The difference of photocurrent between linear and circular polarization excitations is due to the anisotropic response of Te as shown in Fig. 2h, as a result of the anisotropic absorption^45,46^.

**Fig. 3:**
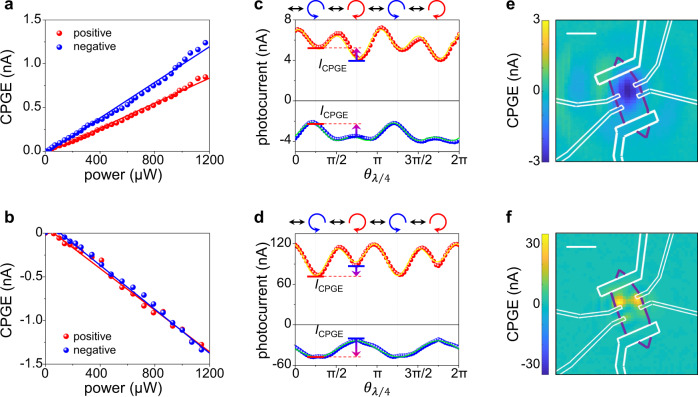
Power-dependent and spatial-resolved CPGE in Te. **a** Power-dependent CPGE under 10.6-μm excitation at positions with the maximal positive (red) and negative (blue) responses on another Te device. **c** ${\theta }_{\lambda /4}$-dependent photocurrent under 10.6-μm excitation at positions with the maximal positive (red) and negative (blue) responses on the T**e** device. **e** The spatial-resolved CPGE under 10.6-μm excitation on the Te device. The scale bar is 10 μm. **b**, **d**, **f** Experiments carried out under 4.0-μm excitation.

In the next, we demonstrate experimentally that the observed CPGE should arise from an intrinsic second-order nonlinear injection current response. Firstly, mirror symmetries are absent in Te so that in-plane second-order effects are non-vanishing under normal incidence excitation. Secondly, this second-order nonlinear optical effect should have a linear dependence on the incident power, which is confirmed by power-dependent CPGE measurements. The intensity of CPGE is obtained by subtracting the photoresponse under the LCP and the RCP excitation at positions with the strongest positive and negative photoresponse, respectively. As shown in Fig. 3a, b, the CPGE shows exactly linear power dependence under 10.6-μm and 4.0-μm excitation (see Supplementary Section 5 for power-dependent photocurrent in Te).

However, the linear power dependence alone is insufficient to confirm the observed optical response is a second-order nonlinear effect, as it cannot rule out the third-order effect that involves a DC electric field (${{{{{{\bf{E}}}}}}}_{{{{{{\rm{DC}}}}}}}$). Such third-order nonlinear injection currents were reported to exhibit linear excitation power dependence in our previous work on type-II Weyl semimetal TaIrTe_4_ and MoTe_2_^27,31^. This third-order effect is assisted by a ${{{{{{\bf{E}}}}}}}_{{{{{{\rm{DC}}}}}}}$ that is independent on the incident light. In the cases of MoTe_2_ and TaIrTe_4_, the built-in electric fields near semimetal-metal contacts play the role of ${{{{{{\bf{E}}}}}}}_{{{{{{\rm{DC}}}}}}}$. The directions of ${{{{{{\bf{E}}}}}}}_{{{{{{\rm{DC}}}}}}}$ are opposite at two contact interfaces in a two-terminal device, so the third-order CPGE response switches sign at the two contact interfaces accordingly. Consequently, the sign of such third-order CPGE response relies on the direction of ${{{{{{\bf{E}}}}}}}_{{{{{{\rm{DC}}}}}}}$. This is different from a second-order CPGE response whose sign is independent on ${{{{{{\bf{E}}}}}}}_{{{{{{\rm{DC}}}}}}}$. Experimentally, we carried out ${\theta }_{\lambda /4}$-dependent measurement at different Te-metal contacts marked by the red and the blue circles in Fig. 2e, where directions of ${{{{{{\bf{E}}}}}}}_{{{{{{\rm{DC}}}}}}}$ should be opposite. The measurement results clearly indicate the CPGE is along the same direction with both 10.6-μm (Fig. 3c) and 4.0-μm (Fig. 3d) excitations at the two contacts. In addition, scanning photocurrent measurements with LCP and RCP excitations are performed to explore the sign of CPGE over the whole device (see Supplementary Fig. 11 for scanning photocurrent mappings). The distribution of CPGE over the device can be obtained by subtracting the photocurrent mapping under the LCP from that under the RCP excitations. As shown in Fig. 3e, f, the sign of CPGE is the same over the whole device. This further confirms that the observed CPGE is a second-order nonlinear optical effect and rules out the possibility of third-order response that is assisted by **E**_DC_. In addition, we note the CPGE in Te is also much stronger than that in other layered Weyl semimetals, such as TaIrTe_4_ and MoTe_2_^27,28,31^, where the generation of a non-vanishing CPGE relies on external factors such as a built-in electric field^27,31^ or the gradient of light intensity^28^. Furthermore, other mechanisms, which may lead to helicity-dependent photocurrent, such as circular photon drag effects^47^ and a chiral edge photocurrent^48^ et al, can also be ruled out according to the measurement geometry and experimental results (see Supplementary Section 7).

**Fig. 4:**
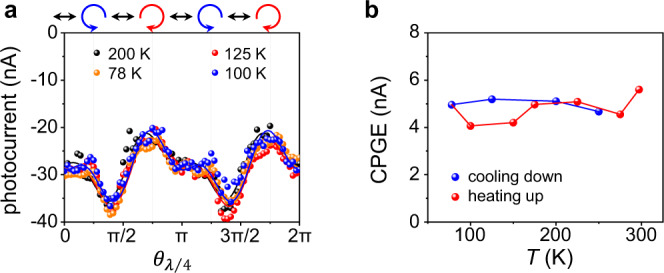
Temperature-dependent CPGE of Te. **a** ${\theta }_{\lambda /4}$-dependent photocurrent at different temperature. **b** Temperature-dependent CPGE amplitude of the Te device. Photocurrent was measured under 4.0-μm excitation of 500 μW.

### Semiconducting band gap of Te

It should be noted that the interpretation of the experimental results under 4.0-μm excitation is based on that transition 2$\to$3 is above the bandgap. Since the transition of 4.0-μm (0.31 eV) excitation is right at the band edge and there are discrepancies about the bandgap for tellurium samples of different morphologies grown by different methods in the literature, it is questionable whether the 4.0-μm excitation corresponds to a transition above the bandgap. Previously, a bandgap of 0.38, 0.35 and 0.335 (or 0.32) eV has been reported in Te crystals^11^, thin films^42^ and bulk^40,41^, respectively. Optical absorption measurements have given a gap value of 0.31 eV and temperature-dependent electrical measurements have provided a smaller gap value of 0.28 eV in Te nanoflakes^10^. Some of these results support the above-bandgap transitions under 4.0-μm excitation. Since the bandgap is temperature dependent, and the bandgap of Te increases by about 10 meV when cooled down from room temperature to 77 K according to the previous experiments^8^,$\,{\theta }_{\lambda /4}$-dependent photocurrent measurement under 4-μm excitation is performed at different temperatures as shown in Fig. 4a. The extracted CPGE components are highly stable during a complete cooling and heating cycle (Fig. 4b). The stable photocurrent response indicates that the transition under 4.0-μm excitation is still above-gap even at 77 K when the bandgap increases compared to that at room temperature (see Supplementary Section 8 for other experimental results about bandgap). In the Supplementary Information, gate-voltage-dependent CPGE measurements are also attempted (see Supplementary Section 9), the strength of CPGE can be tuned by a back gate in a wide range, however the gate tuning effect cannot be quantitatively interpreted due to the interplay of multiple effects induced by single gate tuning, especially the simultaneous tuning of the Fermi level and the band structures^40,49,50^.

In summary, the sign reversal of CPGE response at different mid-infrared wavelengths provides an unambiguous signature for optical transitions related to spin-spitting bands in Te and strictly follows the unique chiral optical selection rules. The two kinds of helicity-dependent optical transitions occur in a single Weyl cone in valence bands and between Weyl cones across the semiconducting gap, respectively. Our optical measurements are consistent with the character that Weyl cones and band gap coexist in Te and help establish Te as a “Weyl semiconductor”, which combines the advantages of topological characteristics of Weyl cones and the highly tunable and controllable nature of a semiconductor. Together with an ultrahigh mobility^10,51^, the strain- and thickness-tunable bandgap^8,40,42,50^, the two-dimensional layered structure and the excellent air-stability^51,52^, Te not only provides an ideal platform for exploring and manipulating exotic topological physics in semiconducting materials, but also promises unprecedent applications toward versatile functional Weyl devices.

## Methods

### Sample growth and device fabrication

Te flakes were synthesized by hydrothermal method. 1.5 g of polyvinylpyrrolidone (molecular weight = 58,000) and 0.046 g of Na_2_TeO_3_ were dissolved in 16 mL of deionized water in sequence with continuous stirring until the solution became clear. The mixed solution was transferred into a 25 mL Teflon-lined stainless steel autoclave filled with ammonium hydroxide solution and hydrazine monohydrate and then heated at 180 °C for 10 h in an oven. The reaction product was washed in deionized water several times by centrifugation to remove residual ions. After purification, the final obtained Te flakes were redispersed in ethanol. The thickness of the as-grown Te flakes were ranging from 20 to 70 nm. To fabricate the Hall-bar device, Te flakes were first transferred onto 285 nm SiO_2_/Si substrate through a drop-casting process, then the electrodes were defined on Te flakes using electron beam lithography, followed by the deposition of Ti/Pd/Au (0.5/20/70 nm) using electron beam evaporation.

### Photocurrent measurement

Mid-infrared photoresponse measurement was carried out with continuous wave quantum cascade lasers centered at 10.6 and 4.0 μm, respectively. A 40X reflective objective lens was used to focus the light to around 20 and 10 μm in spot size for these two wavelengths, respectively. Photocurrent was collected under zero-bias voltage mode. Reflected light was collected and recorded by a commercial LN2-cooling HgCdTe photodetector and PbSe biased photodetector, respectively. For spatial-resolved photocurrent measurement, the devices were placed on a two-dimensional stage which is controlled electrically for scanning photocurrent and reflection images measurement. For polarization-dependent photocurrent measurement, a linear polarizer was set to ensure the linear polarization of incident light. Quarter wave-plates for 10.6, 4.5, and 4.0 μm were rotated electrically to obtain circular polarizations and photocurrent is recorded as a function of angle of QWPL (${\theta }_{\lambda /4}$).

## Supplementary information

Supplementary Information

## Data Availability

All data that support the plots within this paper are available via the figshare repository [10.6084/m9.figshare.20961091.v1].
