# Supplementary material for: Unveiling Weyl-related optical responses in semiconducting tellurium by mid-infrared circular photogalvanic effect
Source: Nat Commun. 2022 Sep 15;13:5425. doi: 10.1038/s41467-022-33190-3 (PMC9477843; doi:10.1038/s41467-022-33190-3)
Supplement: Supplementary file 1 — Supplementary Information [file 41467_2022_33190_MOESM1_ESM.pdf]

Supplementary information
for
Unveiling Weyl-related optical responses in semiconducting tellurium by mid-infrared circular photogalvanic effect

Junchao Ma[1], Bin Cheng[2,3,4], Lin Li[2,3,4], Zipu Fan[1], Haimen Mu[2,3], Jiawei Lai[1], Xiaoming Song[1,5], Dehong Yang[1], Jinluo Cheng[6], Zhengfei Wang[2,3], Changgan Zeng[2,3,4,†], Dong Sun[1,7,†]

[1]International Center for Quantum Materials, School of Physics, Peking University, Beijing 100871, P. R. China

[2]International Center for Quantum Design of Functional Materials, Hefei National Laboratory for Physical Sciences at the Microscale, University of Science and Technology of China, Hefei, Anhui 230026, P. R. China

[3]Synergetic Innovation Center of Quantum Information & Quantum Physics, University of Science and Technology of China, Hefei, Anhui 230026, P. R. China

[4]Chinese Academy of Sciences Key Laboratory of Strongly Coupled Quantum Matter Physics, Department of Physics, University of Science and Technology of China, Hefei, Anhui 230026, P. R. China

[5]State Key Laboratory of Precision Measurement Technology and Instruments, School of Precision Instruments and Opto-electronics Engineering, Tianjin University, Tianjin 300072, P. R. China.

[6]Changchun Institute of Optics, Fine Mechanics and Physics, Chinese Academy of Sciences, Changchun 130033, China.

[7]Collaborative Innovation Center of Quantum Matter, Beijing 100871, China.

[†]email: cgzeng@ustc.edu.cn (C.G.Z); sundong@pku.edu.cn (D.S.)

This Supplemental information includes Supplementary Section 1-9 and Supplementary Figure 1-15.

Contents:

S1. Temperature-dependent resistance of tellurium

S2. Numerical simulations of CPGE responses

S3. Fourier transform of $\theta_{\lambda/4}$-dependent photocurrent response

S4. Circular photogalvanic effect on other tellurium devices

S5. Power-dependence of circular photogalvanic effect

S6. Scanning photocurrent images under different excitation polarizations

S7. Alternative helicity-dependent photocurrent generation mechanisms

S8. Fourier transform infrared spectroscopy of tellurium

S9. Gate-voltage-dependent circular photogalvanic effect of tellurium

Supplementary Section 1. Temperature-dependent resistance of tellurium

Temperature-dependent resistance of a typical tellurium (Te) device is shown in Supplementary Fig. 1, revealing typical characters of a doped semiconductor. As the temperature decreases, the resistance decreases first and then increases, reaching a minimum at $T = 30$ K. Below 30 K, phonon scattering no long dominates. The increase of resistance can be ascribed to the carrier freeze-out effect, which is a common behavior for semiconductors at low temperature.

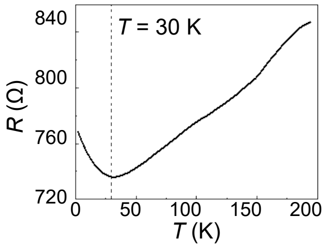

Supplementary Fig. 1 Temperature-dependent resistance of Te.

Supplementary Section 2. Numerical simulations of CPGE responses

In this section, we use two different numerical approaches to show that sign reversal of CPGE at 4.0 μm and 10.6 μm excitation is consistent with optical selection rules determined by band structures in Te. In the numerical calculations of the both approaches, a tight-binding Hamiltonian is used to reproduce the electronic eigenstates, energy eigenvalues and to calculate velocity operators. The Hamiltonian is obtained from the band fitting to the first-principles energy bands with spin-orbital coupling by using the Wannier90 package [1]. The p_x, p_y, p_z three orbitals of Te are used in the band fitting, which are sufficient to make almost the same bands as the first-principles results.

Calculation of optical matrix elements is vital to determine the optical selection rules because the typical ones determined by two-band Hamiltonian of a single Weyl cone (as shown in Fig. 2a of the main text) fails in Te for the following three reasons. Firstly, both spin-splitting valence and conduction bands are formed by strong spin-orbit coupling in Te, so that spin angular momentum of specific energy band is not a good quantum number and cannot get a fixed value. Secondly, because the total angular momentum of photons and electrons must be conserved, orbital angular momentum has an impact on helicity-dependent optical selection rules. Large orbital angular momentum of electrons [2] on specific energy band must be considered when circularly-polarized light is incident to the system. Thirdly, transitions 2→3 and 2→4 cross the band gap and definitely goes beyond two-band Hamiltonian of a single Weyl cone. By calculating optical matrix elements for circularly polarized light, we can comprehensively consider factors above and directly obtain the probability of transitions caused by left or right circular polarization.

S 2.1. Optical matrix element of interband transitions between different energy bands of tellurium

In this section, we determine the helicity-dependent optical selection rules in Te by

calculating optical matrix elements for interband transitions induced by circularly polarized light. Optical matrix elements can be calculated following equation S1:

$$v_{vc}^{\alpha}(\mathbf{k}) = \langle u_{v\mathbf{k}} | \widehat{v^{\alpha}} | u_{c\mathbf{k}} \rangle \tag{S1}$$

where $|u_{v(c)\mathbf{k}}\rangle$ is the electronic eigenfunction at momentum $\mathbf{k}$ of the valence (conduction) band; $\widehat{v^{\alpha}}$ denotes to velocity operator along direction α and is given by:

$$\widehat{v^{\alpha}} = \frac{1}{\hbar}\frac{\partial \hat{H}}{\partial k^{\alpha}} \tag{S2}$$

Considering that interband transitions are induced by the left and right circularly polarized light, and that the wave vector of light is along crystallographic y-axis in our experiment, the optical matrix elements should be written as equation S3:

$$v_{vc}^{\pm}(\mathbf{k}) = v_{vc}^{x}(\mathbf{k}) \pm i v_{vc}^{z}(\mathbf{k}) \tag{S3}$$

According to equation S3, we calculated optical matrix elements along the L_2-H-L in Brillouin zone as shown in Supplementary Fig. 2. The blue and red curves show calculated values with left- and right-circularly polarized excitation, respectively. When interband transitions occur from band 1 to 2 (1→2) near the H point (Supplementary Fig. 2a), we find that optical matrix elements along the H-L direction are larger with the left-circular polarized excitation, while transitions show no preference between the left- and right-circular polarization along the L_2-H direction. To sum up, the left-circular polarized excitation is more favorable over right-circular polarized excitation to generate electrons along the H-L direction for transition 1→2. In our experiment, the transition 1→2 can be realized with 10.6-μm excitation.

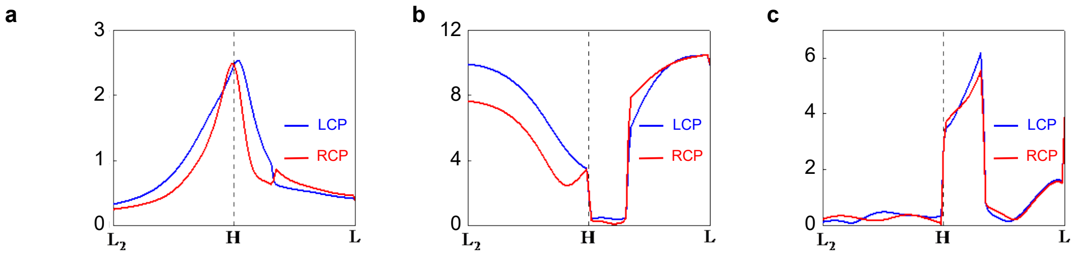

Supplementary Fig. 2 Optical matrix elements of interband transitions of Te for interband transitions. a 1→2 and **b** 2→3 and **c** 2→4.

Still along the L_2-H-L in Brillouin zone, we also calculate the optical matrix element when interband transitions occur from band 2 to 3 (2→3) near the H point (Supplementary Fig. 2b). When transitions happen along the H-L direction, optical matrix elements are similar for left- and right-circular polarized excitation, showing little preference between excitation with opposite helicity. When interband transitions occur near the H point along the opposite L_2-H direction, we find that the transition 2→3 has much larger optical matrix elements with left-circular polarized excitation. As a result, the left-circular polarized excitation induces electrons preferably along L_2-H direction. In our experiment, the transition 2→3 can be realized with 4.0-μm excitation.

The above calculated results imply that, during interband transitions 1→2 and 2→3, electrons are always more likely to be generated with left-circularly polarized light while flow along the opposite directions. Subsequently, opposite CPGE is predicted to be obtained with 10.6-μm and 4.0-μm excitation, respectively. The calculated optical selection rules are shown in Fig. 2b of the main text, and sign reversal of CPGE with 10.6-μm and 4.0-μm excitation in our experiments is consistent with these selection rules. Additionally, 4.0-μm excitation may also induce optical transition from band 2 to 4 (2→4). Calculated optical matrix elements show little preference between the left- and right-circular polarizations along the L_2-H-L (Supplementary Fig. 2c). This result won't affect the consistency of calculations and experiments in our work.

S 2.2. Tensor elements of injection current

In this section, we verify the sign reversal of CPGE by calculating tensor elements of injection current with 10.6-μm and 4.0-μm excitations, respectively. This approach can provide the strength of CPGE quantitively. In the calculation, we also consider a cubic region rather than a single direction in Brillouin zone. As a result, this is a more direct and rigorous approach than calculating optical matrix elements.

Firstly, we determine the expression of the nonlinear photocurrent by symmetry analysis. Considering the symmetry of lattice, Te belongs to D_3 point group. For optical electric field in *x*-*z* plane, nonzero tensor elements are σ^{yzx} and σ^{yxz}. The relationship between these two elements is:

$$\sigma^{yzx}(\omega,-\omega) = s_{\mathrm{r}} + \mathrm{i}s_{\mathrm{i}} \tag{S4}$$
$$\sigma^{yzx}(-\omega,\omega) = s_{\mathrm{r}} - \mathrm{i}s_{\mathrm{i}} \tag{S5}$$
$$\sigma^{yxz}(-\omega,\omega) = s_{\mathrm{r}} + \mathrm{i}s_{\mathrm{i}} \tag{S6}$$
$$\sigma^{yxz}(\omega,-\omega) = s_{\mathrm{r}} - \mathrm{i}s_{\mathrm{i}} \tag{S7}$$

s_{r} and s_{i} denotes to the real and imaginary part of nonlinear tensor elements, respectively. Consequently, direct photocurrent generated from second order nonlinear effects can be obtained as:

$$J(0) = \sigma^{yxz}(\omega,-\omega)E^x(\omega)E^z(-\omega) + \sigma^{yzx}(\omega,-\omega)E^z(\omega)E^x(-\omega) + c.c. \tag{S8}$$

According to equation S4 and S7, the generated nonlinear photocurrent is determined by:

$$J(0) = 4s_{\mathrm{r}}\mathrm{Re}[E^z(\omega)E^x(-\omega)] - 4s_{\mathrm{i}}\mathrm{Im}[E^z(\omega)E^x(-\omega)] \tag{S9}$$

Secondly, we extract the CPGE responses from the total nonlinear photocurrent based on the dependence on the rotating angle of the quarter wave plate. Considering the fast axis of a quarter wave-plate is initially set along crystallographic a-axis and the wave plate is rotated continuously, the electric fields of the incident light follow:

$$E_{\mathrm{inc}} = E_0 \mathrm{e}^{\mathrm{i}\frac{\pi}{4}} \begin{pmatrix} \cos^2\theta + \mathrm{i}\sin^2\theta \\ 0 \\ (1-\mathrm{i})\sin\theta\cos\theta \end{pmatrix} \tag{S10}$$

Here, E_0 is the amplitude of incident light field, θ is the angle between the fast axis

of quarter wave-plate and the crystallographic a-axis. Substitute E^x and E^z from equation S10 into equation S9, and the nonlinear photocurrent can be given by

$$J(0) = E_0^2[s_\mathrm{r}\cos 4\theta + 2s_\mathrm{i}\sin 2\theta] \quad \text{(S11)}$$

Considering that both left- and right-circular polarizations occur once when θ changes by 180 degrees, the CPGE responses we observed should correspond to the photocurrent component with a 180-degree period, namely $2s_\mathrm{i}E_0^2\sin 2\theta$. The other photocurrent component has a 90-degree period, namely $s_\mathrm{r}E_0^2\sin 4\theta$. Considering that the switch between a linear polarization and a circular polarization occurs once when θ changes by 90 degrees, the s_r term contributes the linear polarization dependent photocurrent response, while the CPGE responses are contributed by imaginary part of nonlinear photocurrent tensors only.

In a specific non-magnetic system such as Te, CPGE is generated by injection current effect, which is usually identified by an injection coefficient which relates to the conductivity as $\sigma^{yzx} \propto \eta^{yzx}$, and the injection coefficient is given by:

$$\eta^{yzx} = 2\pi\left(\frac{e}{\hbar\omega}\right)^2 \int \frac{\mathrm{d}\mathbf{k}}{(2\pi)^3} \Sigma_{cv}\left(v_{cc}^y - v_{cc}^y\right) v_{vc}^z v_{cv}^x\, \delta(\omega - \omega_{cv}) \quad \text{(S12)}$$

Because this system is non-magnetic, we apply time reversal symmetry to both sides of equation S12 and find out that $\eta_{yzx} = -\eta_{yzx}^*$, which means η_{yzx} is a pure imaginary number. This is consistent with above analysis according to equation S11 in the previous paragraph and thus with our experiment. Namely, it is pure-imaginary injection coefficient that induces CPGE responses, which has a 180-degree period when the quarter wave-plate is rotated in our experiments.

Thirdly, we calculate the relevant tensor elements. Instead of numerically evaluating equation S12 directly, we consider the crystal symmetry and transform equation S12 into the following expression:

$$\eta^{yzx} = \mathrm{i}\pi\left(\frac{e}{\hbar\omega}\right)^2 \int \frac{\mathrm{d}\mathbf{k}}{(2\pi)^3} \Sigma_{cv}\left[\left(v_{cc}^y - v_{vv}^y\right)\mathrm{Im}[v_{cv}^x v_{vc}^z] - \left(v_{cc}^x - v_{vv}^x\right)\mathrm{Im}[v_{cv}^y v_{vc}^z]\right] \delta(\omega - \omega_{cv}) \quad \text{(S13)}$$

Here, $v_{ab}^{\mathbf{k}}$ denotes to velocity matrix elements along direction **k**; *a* and *b* are index of energy bands, and *c* and *v* denotes to label empty and occupied states, respectively; ω_{cv} denotes to the energy difference between the conduction and valence bands at specific position in the momentum space. Both $v_{ab}^{\mathbf{k}}$ and ω_{cv} depend on momentum **k**.

Considering the band edges appear at H_1 and H_2 points in the Brillouin zone, and they are connected by the transformation $y \to -y$ and $z \to -z$ as shown in Supplementary Fig. 3, the integrals in equation S13 near H_1 and H_2 are the same, so that we only need to calculate the imaginary part of the integral near a single H point. The integral is calculated in a small cubic of the momentum space with the H point as the center and 0.1 $\mathrm{\AA}^{-1}$ as the side length. $\delta(\omega - \omega_{cv})$ is replaced with Gaussian

function $\frac{1}{\Delta\sqrt{\pi}}e^{-\frac{(\omega-\omega_{cv})^2}{\Delta^2}}$, and the broadening Δ is set as 0.1 eV.

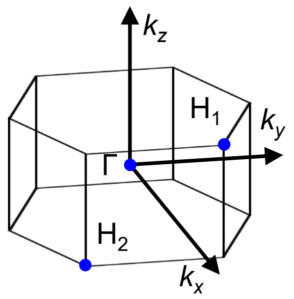

Supplementary Fig. 3 Brillouin zone of Te.

We first calculate tensor elements with 10.6-μm excitation. In this case, only transition 1→2 is relevant and the calculation gives $\mathrm{Im}(\eta^{yzx}_{10.6\ \mu\mathrm{m}}) \sim -2.3 \times 10^{-5}$. Then we calculate tensor elements with 4.0-μm excitation. Both transitions 2→3 and 2→4 are relevant and the imaginary part of tensor elements are $\sim 6.8 \times 10^{-5}$ and $\sim 6.5 \times 10^{-5}$, respectively. The calculation gives $\mathrm{Im}(\eta^{yzx}_{4.0\ \mu\mathrm{m}}) \sim 13.3 \times 10^{-5}$. The sign reversal of CPGE with 10.6-μm and 4.0-μm excitation in our experiments is consistent with calculated tensor elements of injection current.

Supplementary Section 3. Fourier transform of $\theta_{\lambda/4}$-dependent photocurrent response

Photocurrent response under 10.6-μm excitation in Fig. 2f of maintext shows complicated polarization dependence when the angle of quarter wave-plate ($\theta_{\lambda/4}$) is tuned continuously. Fourier transform is applied to distinguish photocurrent with different $\theta_{\lambda/4}$-period as shown in Supplementary Fig. 4a. Major peaks are observed at angular frequencies of 0, 1/π, and 2/π, which are attributed to polarization-independent, circular photogalvanic effect (CPGE), and anisotropic responses, respectively. The photocurrent components of different periodicities are plotted separately in Supplementary Fig. 4b. The CPGE response reaches local peaks under circular polarization excitation as marked by A and B. If we add polarization-independent, CPGE and anisotropic responses together, it recovers the experimentally measured signal plotted by the solid line in Fig. 2f of maintext. The peaks will shift from A and B towards A and B (the local peaks of 2/π-periodicity component), leading to uneven distribution of photocurrent peaks. Fourier transform of photocurrent under 4.0-μm excitation in Fig. 2g of maintext is shown in Supplementary Fig. 4c and 4d.

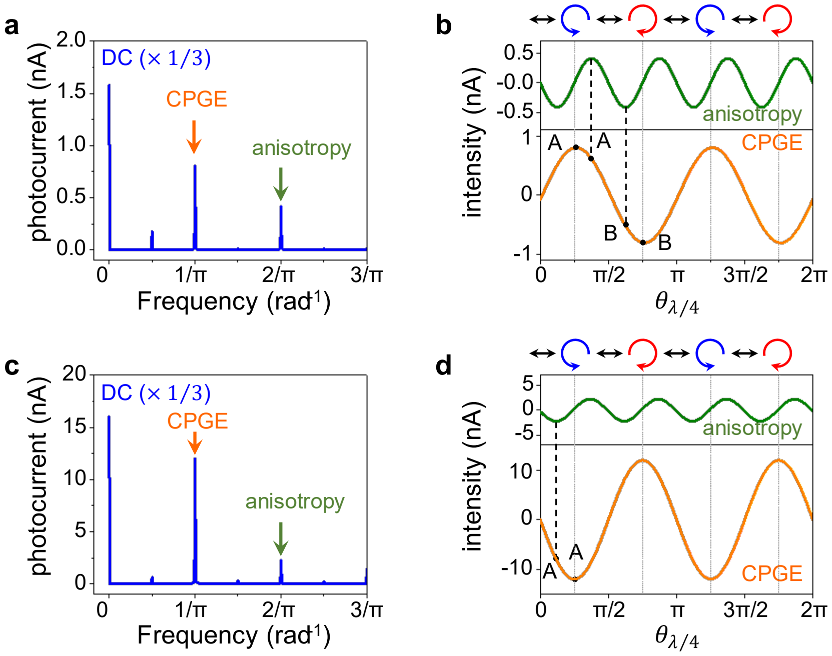

Supplementary Fig. 4 Fourier transform of $\boldsymbol{\theta_{\lambda/4}}$-dependent photocurrent. a Fourier transform of the $\theta_{\lambda/4}$-dependent photocurrent response under 10.6-μm excitation in Fig. 2f of maintext. The polarization-independent component is multiplied by 1/3. **b** The CPGE and anisotropy responses extracted from (**a**). A and B mark local peaks of CPGE. A and B mark local peaks of anisotropy. **c** Fourier transform of the $\theta_{\lambda/4}$-dependent photocurrent response under 4.0-μm excitation in Fig. 2g of maintext. **d** The CPGE and anisotropy responses extracted from (**c**). A and A mark local peaks of CPGE and anisotropy, respectively. The arrows on the top of the panels label the polarization sequences, and the blue and red circles represent left and right circularly polarizations (LCP and RCP), respectively.

Supplementary Section 4. Circular photogalvanic effect on other tellurium devices

S4.1. Results of device 2

For device 2, the CPGE responses along crystallographic *a*-axis show the same characteristics as those shown in the maintext. Electrodes A and B are connected for the current measurements and the other four electrodes are floated. Both spatial-resolved and polarization-dependent photocurrent responses are measured as shown in Supplementary Fig. 5. The signs of CPGE are opposite under 10.6-μm and 4.0-μm excitation, consistent with chirality selection rules. The signs are the same at two sample-metal contact interfaces, confirming the second order CPGE response. All the photocurrent response features are similar to those of the device presented in the maintext.

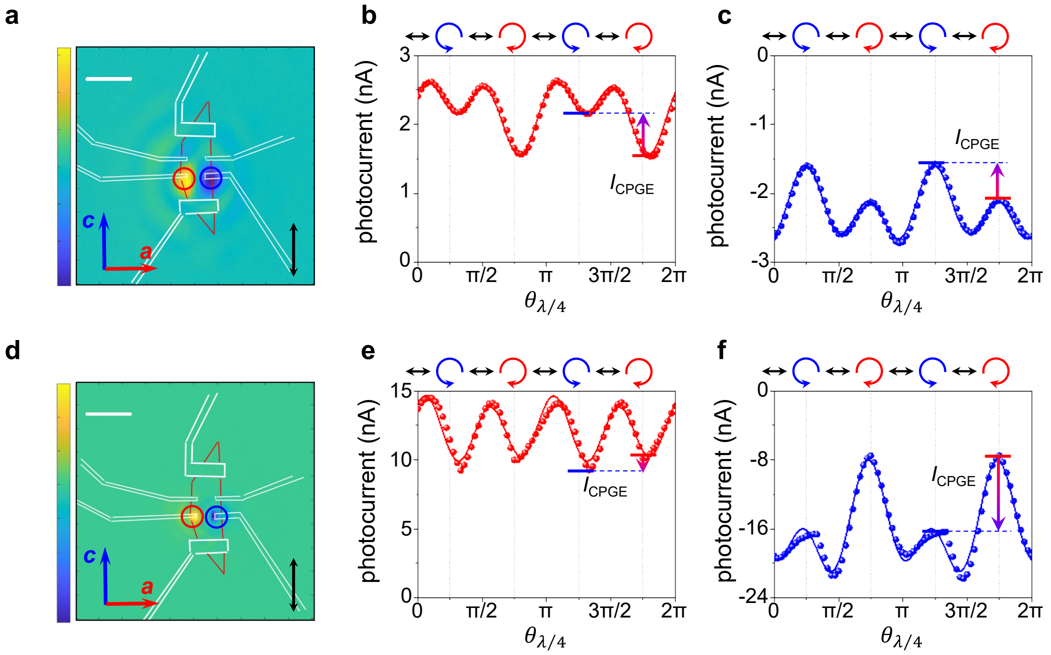

Supplementary Fig. 5 CPGE of device 2 measured using electrodes A and B under 10.6-μm and 4.0-μm excitation. a The scanning photocurrent image of device 2 under 10.6-μm excitation. A and B mark the electrodes used in the measurement and the other four electrodes are floated. The red and blue arrows mark the directions of crystallographic a- and c- axes, respectively. The double-arrow marks the direction of excitation light polarization. The scale bar is 20 μm. **b, c,** The $\theta_{\lambda/4}$-dependent photocurrent response under 10.6-μm excitation. The measurements are performed at positions with maximal positive and negative responses on the device as marked by the red and blue circles in (**a**). The arrows on the top of the panels label the polarization sequences, and the blue and red circles represent LCP and RCP, respectively. **d-f** Scanning and $\theta_{\lambda/4}$ -dependent photocurrent responses under 4.0-μm excitation.

In another measurement, another pair of electrodes, C and D, along the crystallographic a-axis, are connected for photocurrent measurement and the other four electrodes are floated. The spatial-resolved and polarization-dependent photocurrent responses are measured as shown in Supplementary Fig. 6. The CPGE responses show the same characteristics as those presented in the maintext.

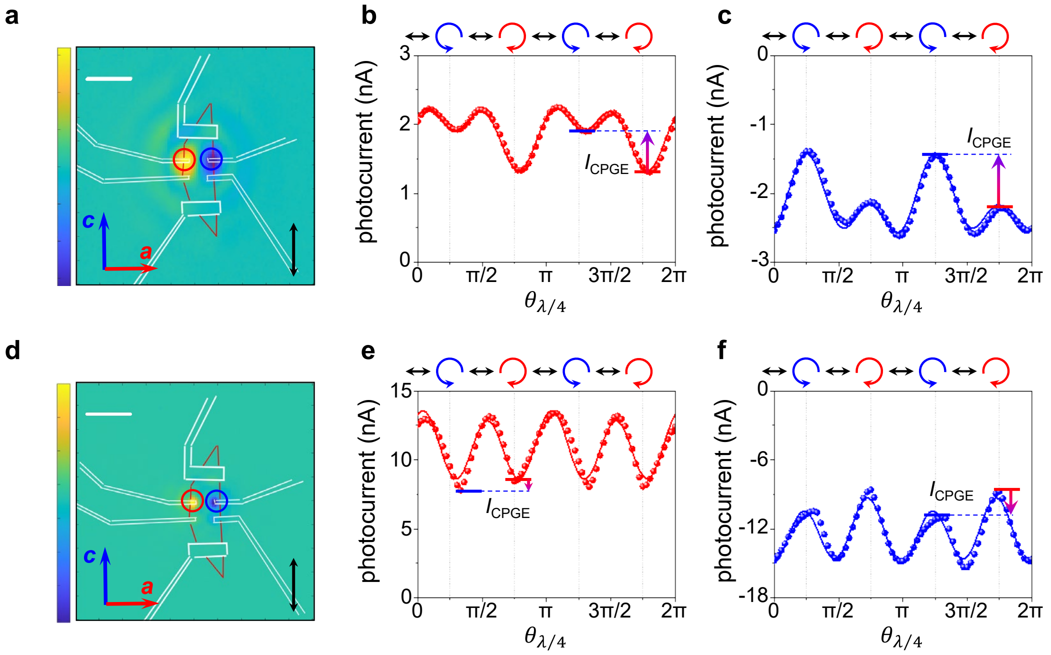

Supplementary Fig. 6 CPGE of device 2 measured using electrodes C and D under 10.6-μm and 4.0-μm excitation. a The scanning photocurrent image of device 2 under 10.6-μm excitation. C and D mark the electrodes used in the measurement and the other four electrodes are floated. The red and blue arrows mark the directions of crystallographic *a*- and *c*-axes, respectively. The double-arrow marks the direction of excitation light polarization. The scale bar is 20 μm. **b, c** The $\theta_{\lambda/4}$-dependent photocurrent response under 10.6-μm excitation. The measurements are performed at positions with maximal positive and negative responses on the device as marked by the red and blue circles in (**a**). The arrows on the top of the panels label the polarization sequences, and the blue and red circles represent LCP and RCP, respectively. **d-f** Scanning and $\theta_{\lambda/4}$ -dependent photocurrent responses under 4.0-μm excitation.

S4.2. Results of device 3

For device 3, the CPGE responses also show the same characteristics as those presented in the maintext (Supplementary Fig. 7 and 8).

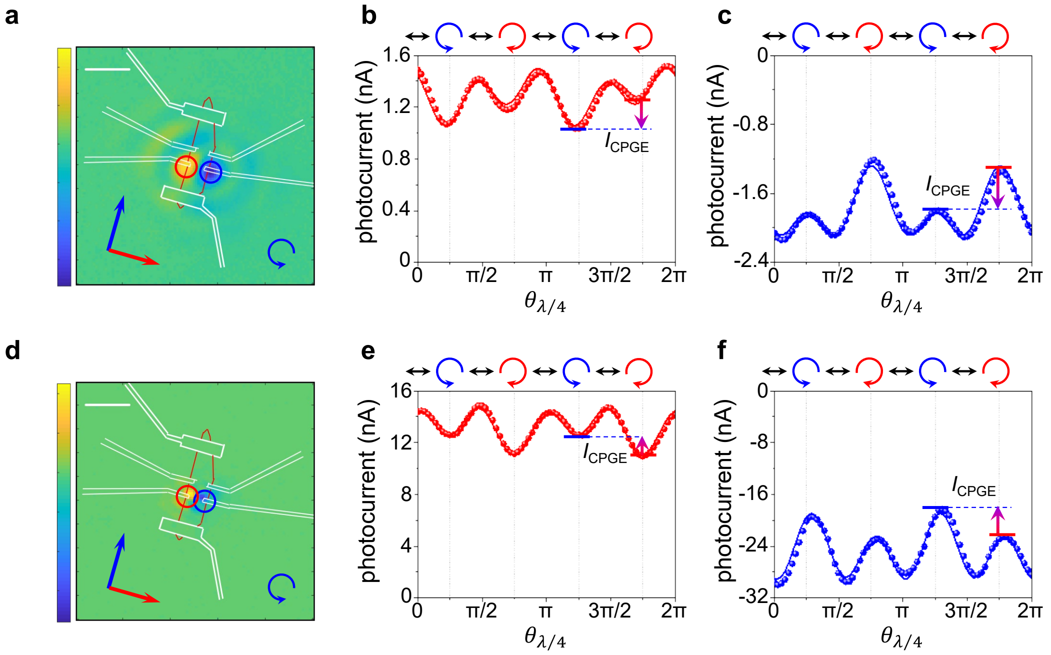

Supplementary Fig. 7 CPGE of device 3 measured using electrodes A and B under 10.6-μm and 4.0-μm excitation. a The scanning photocurrent image of device 3 under 10.6-μm excitation. A and B mark the electrodes used in the measurement and the other four electrodes are floated. The red and blue arrows mark the directions of crystallographic *a*- and *c*-axes, respectively. The blue circular arrow marks LCP. The scale bar is 20 μm. **b, c** The $\theta_{\lambda/4}$-dependent photocurrent response under 10.6-μm excitation. The measurements are performed at positions with maximal positive and negative responses on the device as marked by the red and blue circles in (**a**). The arrows on the top of the panels label the polarization sequences, and the blue and red circles represent LCP and RCP, respectively. **d-f** Scanning and $\theta_{\lambda/4}$-dependent photocurrent responses under 4.0 μm excitation.

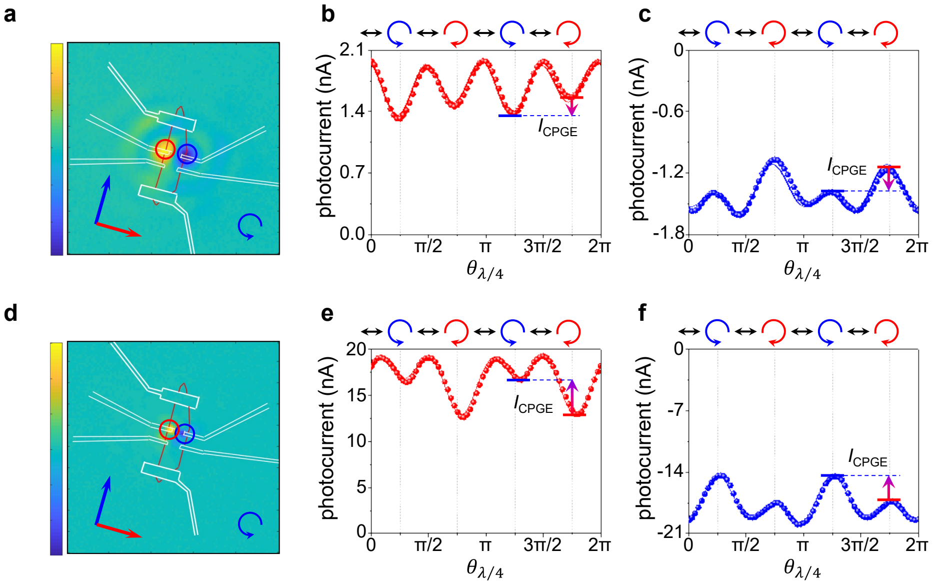

Supplementary Fig. 8 CPGE of device 3 measured using electrodes C and D under 10.6-μm and 4.0-μm excitation. a The scanning photocurrent image of device 3 under 10.6-μm excitation. C and D mark the electrodes used in the measurement and the other four electrodes are floated. The red and blue arrows mark the directions of crystallographic *a*- and *c*-axes, respectively. The blue circular arrow marks LCP. The scale bar is 20 μm. **b, c** The $\theta_{\lambda/4}$-dependent photocurrent response under 10.6-μm excitation. The measurements are performed at positions with maximal positive and negative responses on the device as marked by the red and blue circles in (**a**). The arrows on the top of the panels label the polarization sequences, and the blue and red circles represent LCP and RCP, respectively. **d-f** Scanning and $\theta_{\lambda/4}$-dependent photocurrent responses under 4.0-μm excitation.

Supplementary Section 5. Power-dependence of circular photogalvanic effect

The power-dependent photocurrent is measured on device 2 under 10.6-μm and 4.0-μm excitation as shown in Supplementary Fig. 9a and 9b. LCP and RCP light is focused on positions with maximal positive and negative responses, respectively. The results show linear power dependence at both positions. By subtracting photocurrent responses under RCP and LCP excitation, linear power dependence of CPGE can be obtained as shown in Fig. 3a and 3b of the maintext. The $\theta_{\lambda/4}$-dependent photocurrent measurement with different incident powers is also carried out on device 2, which is shown in Supplementary Fig. 10.

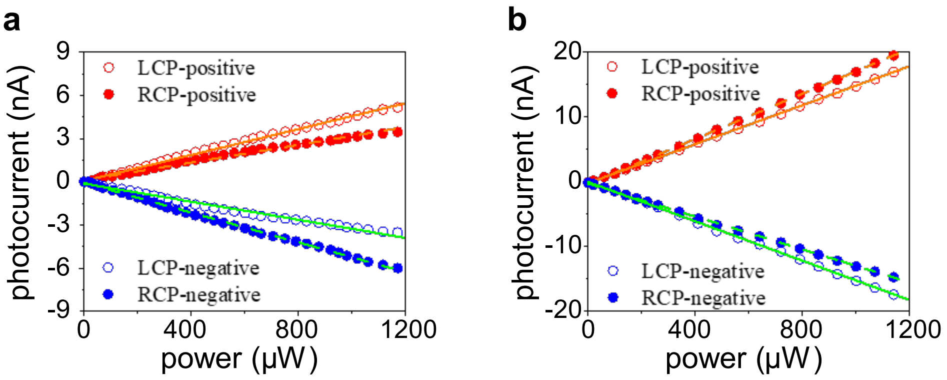

Supplementary Fig. 9 power-dependent photocurrent under LCP and RCP excitation, respectively. a 10.6-μm and **b** 4.0-μm light is focused on positions with maximal positive and negative responses on device 2.

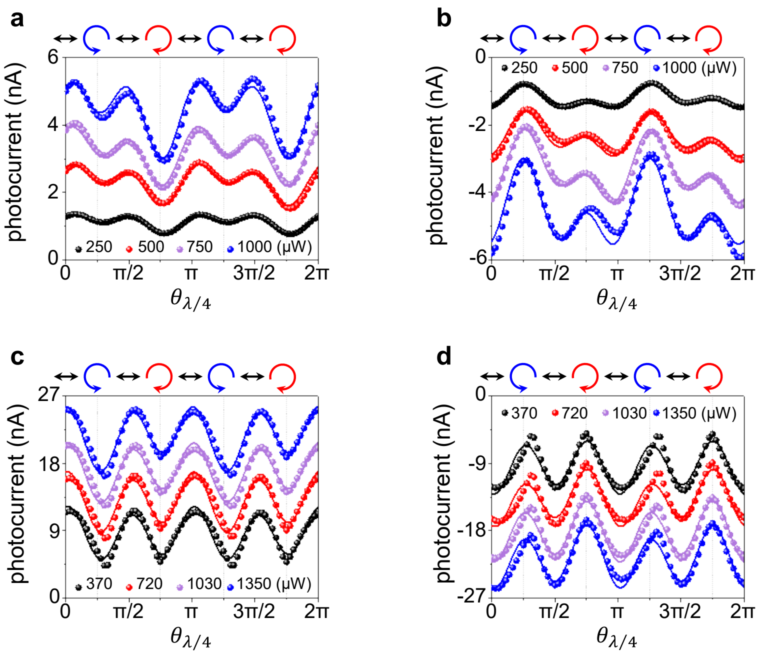

Supplementary Fig. 10 $\boldsymbol{\theta_{\lambda/4}}$**-dependent photocurrent responses with different excitation powers. a, b** $\theta_{\lambda/4}$-dependent photocurrent responses with different excitation powers under 10.6-μm excitation. Light is focused on positions with maximal positive (**a**) and negative (**b**) responses on device 2, respectively. **c, d** $\theta_{\lambda/4}$-dependent photocurrent responses with different excitation powers under 4.0-μm excitation. Light is focused on positions with maximal positive (**c**) and negative **d** responses on device 2, respectively. The arrows on the top of the panels label the polarization sequences, and the blue and red circles represent LCP and RCP, respectively.

Supplementary Section 6. Scanning photocurrent images under different excitation polarizations

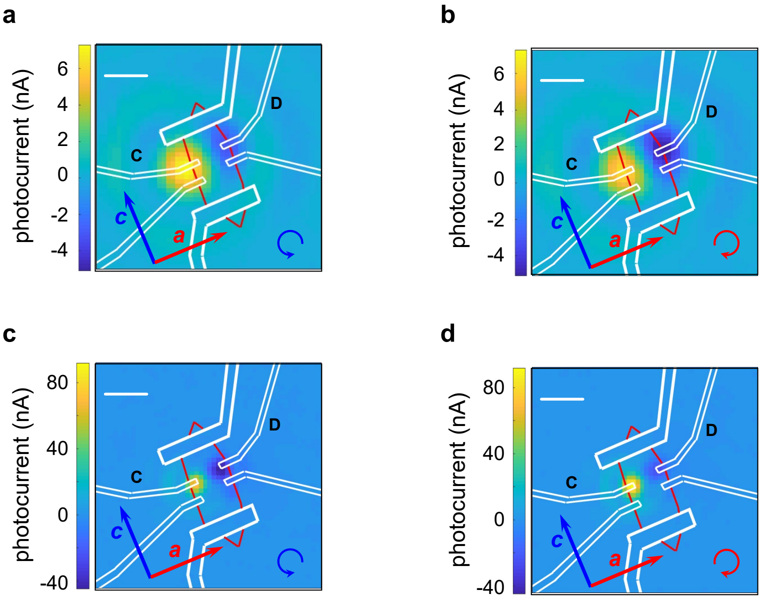

Supplementary Fig. 11 Scanning photocurrent images under different

excitation polarizations. a, b Scanning photocurrent images under 10.6-μm LCP and RCP excitation, respectively. **c, d** Scanning photocurrent images under 4.0-μm LCP and RCP excitation, respectively. C and D mark the electrodes used in the measurement and the other four electrodes are floated. The red and blue arrows mark the directions of crystallographic a- and c-axes, respectively. The blue and red circular arrows represent LCP and RCP, respectively. The scale bars are 10 μm.

Supplementary Section 7. Alternative helicity-dependent photocurrent generation mechanisms

In this section, we discuss alternative helicity-dependent photocurrent generation mechanisms besides transverse CPGE presented in the maintext. We show that we can firmly rule out all other possibilities that may lead to helicity-dependent photocurrent response according to our measurement geometry and experimental results.

S7.1. Circular photon drag effect

In recent studies, circular photon drag effects (CPDE) are reported in Te and can produce transverse helicity-dependent photocurrent, too[3]. However, CPDE links to higher-order nonlinear tensor than CPGE in Te, which means a much smaller response magnitude. We note that third-order nonlinear optical tensor elements only contribute to CPGE, determined by $\chi^{abc}E^{b}E^{c^*}$, while CPDE corresponds to higher-order nonlinear optical tensor elements, determined by $\chi^{abcd}q^{b}E^{c}E^{d^*}$. Considering that rank-4 tensor is much smaller than rank-3 tensor, and q^{b} is rather small for a photon, CPGE should contribute to much stronger (orders larger) signals on the generation of helicity-dependent photocurrent than that from CPDE.

Experimentally, PDE is usually observed in centrosymmetric materials where PGE is forbidden by inversion symmetry. In Te, CPDE can be observed only when a specific experimental geometry was selected carefully to exclude CPDE [3]. With experimental geometry in our work, both CPGE and CPDE can exist. Helicity-dependent photocurrent can reach nearly 10^3 nA/W under 10.6-μm excitation in our work, while the maximum helicity-dependent photocurrent generated by CPDE is only around 10 nA/W with an incidence angle of 10 degrees according to previous measurements in the literature and is much smaller with an incidence angle of 0 degree [3]. So that helicity-dependent photocurrent should mainly originate from CPGE rather than CPDE, and the contribution from CPDE, even exists, is minor comparing to the response from CPGE in our work.

S7.2. Chiral edge currents

An edge photocurrent can stem from lower symmetry near the sample edges. Scattering of carriers driven by the circular polarized field determines the chirality of edge current. This phenomenon was first observed in graphene with THz radiation [4]. In our experiments, helicity-dependent photocurrent distributes at the area between

two electrodes under 10.6-μm excitation, or near the sample-metal contact interfaces under 4.0-μm excitation, instead of the edges of samples. Edge currents should not play a role under the measurement geometry in our work.

S7.3. Photogalvanic effect driven by electron spin

Spin galvanic effect: helicity-dependent photocurrent was proposed and observed in semiconductor quantum well[5] driven by electron spin. Owing to spin-orbit interaction from asymmetric potentials, spin degeneracy of sub-bands can be lifted. Once these sub-bands are shifted in **k** space and inherent asymmetry in spin-flip transitions exists, a helicity-dependent photocurrent can be generated. However, this effect relies on combination of a magnetic field to realize rotation of non-equilibrium spin polarization.

Photo-induced inverse spin Hall effect: helicity-dependent photocurrent was observed in bulk GaAs due to photo-induced inverse spin hall effect[6]. In such effect, spin-dependent scattering happens during diffusion of photo-induced spin-oriented electrons from the surface into the bulk, resulting in observable helicity dependent photocurrent. However, this effect relies on a long spin-lifetime of electrons during the diffusion which does not apply to Te. The spin-degeneracy of conduction bands in GaAs leads to long spin-lifetime, while simultaneous lift of spin-degeneracy of both valence and conduction bands usually results in an extremely short spin-lifetime[7]. We do not expect the photo-induced inverse spin Hall effect should play a vital role in such spin texture.

Supplementary Section 8. Fourier transform infrared spectroscopy of tellurium

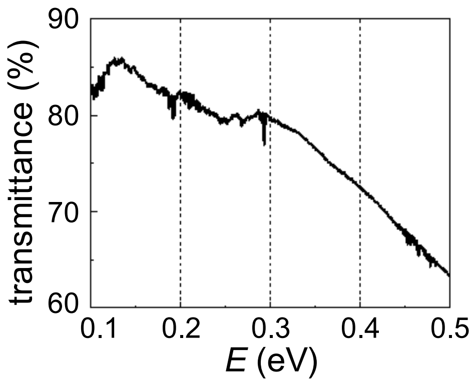

Supplementary Fig. 12 Fourier transform infrared spectroscopy of Te. An unambiguous absorption edge cannot be extracted clearly but absorption behaviors can still be observed at 4.0 μm (0.31 eV) excitations.

Supplementary Section 9. Gate-voltage-dependent circular photogalvanic effect of tellurium

In this session, we measure the circular-polarization-dependent photocurrent under 4.0-μm and 10.6-μm excitations by applying back-gate voltages to tune the doping of Te.

When Fermi level is tuned by an electric gating, it may cause effects on optical transitions due to Pauli blockings. CPGE is expected to vanish or reverse under specific excitation wavelength and with suitable Fermi level. At the same time, the change of doping may affect the carrier densities involved in the optical transitions, which can affect the amplitude of photocurrent responses drastically.

S9.1. Gating effect by transport measurement at different temperatures

In a typical Te transistor, the hole-doping behaviors can be observed through the transfer curve measured at 1.5 K as shown in Supplementary Fig. 13. The chemical neutral point is obtained under a back-gate voltage at around 68.5 V. However, the current on/off ratio will go through a drastic drop and the chemical neutral point is shifted to a higher gate voltage when temperature increases as previously reported in the reference [8]. This is because thermal excitation leads to an increase of carrier densities at higher temperature, especially for narrow-bandgap materials. The thermal activation can provide available initial and final states for transition 1→2 under 10.6 μm and transition 2→3 under 4.0 μm, so that CPGE at specific back-gate voltages can be affected for room temperature measurement.

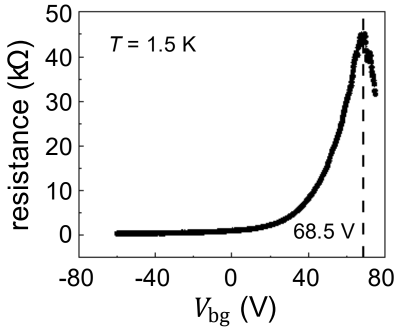

Supplementary Fig. S13 Transfer curves of a typical Te transistor measured at 1.5 K.

S9.2. Gate-voltage-dependent circular photogalvanic effect under 4.0-μm excitation

In our experiment, 4.0-μm excitation can induce the spin-flip transition between two Weyl cones across the bandgap, from energy band 2 to 3 (2→3) as shown in Supplementary Fig. 14a. Ideally, applying a negative gate voltage will lower the Fermi level and lead to blocking of the transition 2→3 and emergence of the transition from energy band 1 to 2 (1→2) as shown in Supplementary Fig. 14a. CPGE should vanish at first, and then recover with a sign reverse.

The experiment is performed on device 4 and the Fermi level can be tuned by back-gate voltages. Electrodes A and B along crystallographic *a*-axis are connected for photocurrent measurements and the other four electrodes are floated. Scanning photocurrent responses under 4.0-μm excitation are measured as shown in Supplementary Fig. 14b. The signs of photocurrent responses are opposite at two sample-metal contact interfaces. $\theta_{\lambda/4}$-dependent photocurrent responses are measured

at positions with maximal positive and negative responses on the device as shown in Supplementary Fig. 14c and 14d. CPGE components extracted by Fourier transform are shown in Supplementary Fig. 14e and 14f. A back-gate voltage between ±84 V only changes the amplitude of CPGE while the expected vanishing and reversal of CPGE doesn't happen.

We speculate the thermal activation at room temperature should account for the CPGE at a high gate voltage right below the damage threshold by contributing available initial and final states for the transition 2→3 under 4.0-μm excitation. On the other hand, the back gating may also induce other effects beyond the modification of Fermi level. For instance, Te is a well-known piezoelectric material and can generate a large piezoelectric strain under an external electric field or incident light[9,10]. This strain can lead to evolution of band structures[11] and the change of band structures can affect the CPGE response. Therefore, the influences of these effects by applying a back gate on the photocurrent responses are complicate and not clear at present, but many of these effects can modify the CPGE response significantly from a simple diagram of Fermi level tuning.

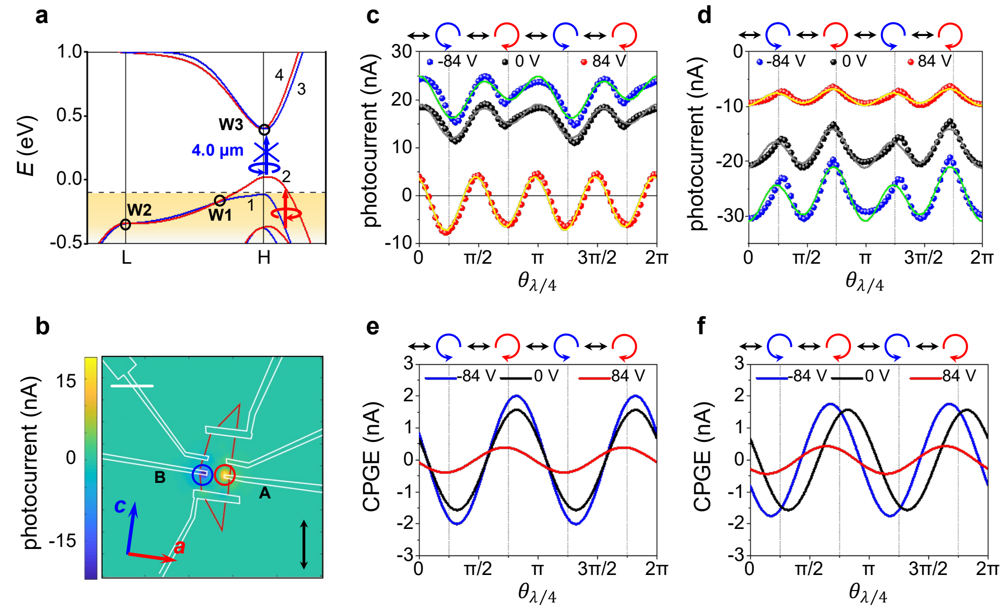

Supplementary Fig. 14 Gate-voltage-dependent CPGE under 4.0-μm excitation. a Band diagram near the H point of Te taking spin-orbit interaction into consideration. The red and blue arrows mark transitions induced by 4.0-μm RCP and LCP excitations with suitable Fermi levels, respectively. W1, W2, and W3 mark three Weyl nodes near H point. Energy bands forming W1 and W3 are marked by 1-4. The dashed line marks the lower Fermi level compared to that without gate voltages. **b** The scanning photocurrent image of device 4. A and B mark the electrodes used in the measurement and the other four electrodes are floated. The red and blue arrows mark the directions of crystallographic *a*- and *c*-axes, respectively. The double-arrow marks the direction of excitation light polarization. The scale bar is 20 μm. **c, d** The $\theta_{\lambda/4}$-dependent photocurrent responses at

different gate voltages. The measurements are performed at positions with maximal positive (**c**) and negative responses (**d**) on the device as marked by the red and blue circles in (**b**), respectively. **e, f** CPGE responses at different gate voltages extracted from (**c**) and (**d**), respectively. The arrows on the top of the panels label the polarization sequences, and the blue and red circles represent LCP and RCP, respectively.

S9.3. Gate-voltage-dependent circular photogalvanic effect under 10.6-μm excitation

In our experiment, 10.6-μm excitation can induce the spin-flip transition 1→2 within the Weyl cone W1 as shown in Supplementary Fig. 15a. Ideally, applying a positive gate voltage will lift Fermi level and lead to blocking of the transition 1→2 and thus the related CPGE should disappear. In the measurement, electrodes C and D are connected for photocurrent measurements and the other four electrodes are floated. Scanning photocurrent responses under 10.6-μm excitation are measured as shown in Supplementary Fig. 15b. The photocurrent response mainly occurs near the electrode C. $\theta_{\lambda/4}$-dependent photocurrent responses are measured at the position with a maximal positive response on the device as shown in Supplementary Fig. 15c. The CPGE components extracted by Fourier transform are shown in Supplementary Fig. 15d. A back-gate voltage between ±106 V only changes the amplitude of CPGE while the expected vanishing of CPGE doesn't happen. Similar to the 4.0-μm excitation case, we speculate the thermal activation at room temperature should account for the CPGE at a high gate voltage by contributing available initial and final states for the transition 1→2 under 10.6-μm excitation. Other effects induced by the back gating beyond the modification of Fermi level can also have complicate contributions to the CPGE responses which remain to be studied in the future.

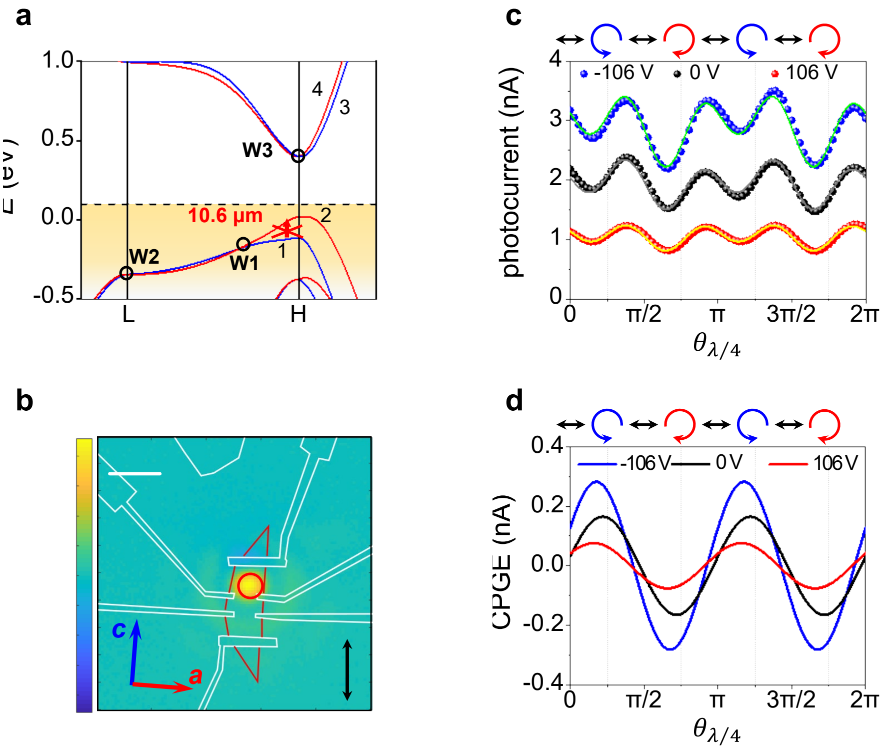

Supplementary Fig. 15 Gate-voltage-dependent CPGE under 10.6-μm excitation. a Band diagram near the H point of Te taking spin-orbit interaction into consideration. The red arrow marks the transition induced by 10.6-μm RCP excitation with suitable Fermi levels. W1, W2, and W3 mark three Weyl nodes near H point. Energy bands forming W1 and W3 are marked by 1-4. The dashed line marks the higher Fermi level compared to that without gate voltages. **b** The scanning photocurrent image of device 4. C and D mark the electrodes used in the measurement and the other four electrodes are floated. The red and blue arrows mark the directions of crystallographic a- and c-axes, respectively. The double-arrow marks the direction of excitation light polarization. The scale bar is 20 μm. **c** The $\theta_{\lambda/4}$-dependent photocurrent responses at different gate voltages. The measurements are performed at the position with a maximal positive response on the device as marked by the red circle in (**b**). **d** CPGE responses at different gate voltages extracted from (**c**). The arrows on the top of the panels label the polarization sequences, and the blue and red circles represent LCP and RCP, respectively.

Supplementary References

1. Mostofi, A. A., Yates, J. R., Pizzi, G. et al. An updated version of wannier90: A tool for obtaining maximally-localised Wannier functions. Computer Physics Communications **185**, 2309-2310 (2014).
2. Tsirkin, S. S., Puente, P. A. & Souza, I. Gyrotropic effects in trigonal tellurium studied from first principles. Phys. Rev. B **97**, 035158 (2018).
3. Shalygin, V. A., Moldavskaya, M. D., Danilov, S. N. et al. Circular photon drag effect in bulk tellurium. Phys. Rev. B **93**, 045207 (2016).
4. Karch, J., Drexler, C., Olbrich, P. et al. Terahertz Radiation Driven Chiral Edge Currents in Graphene. Phys. Rev. Lett. **107**, 276601 (2011).
5. Ganichev, S. D., Ivchenko, E. L., Belkov, V. V. et al. Spin -galvanic effect. Nature **417**, 153-156 (2002).
6. Kikkawa, J. M. & Awschalom, D. D. Resonant Spin Amplification in n-Type GaAs. Phys. Rev. Lett. **80**, 4313-4316 (1998).
7. McIver, J. W., Hsieh, D., Steinberg, H. et al. Control over topological insulator photocurrents with light polarization. Nat. Nanotechnol. **7**, 96-100 (2012).
8. Amani, M., Tan, C., Zhang, G. et al. Solution-Synthesized High-Mobility Tellurium Nanoflakes for Short-Wave Infrared Photodetectors. ACS Nano **12**, 7253-7263 (2018).
9. Hermann, J. P., Quentin, G. & Thuillier, J. M. Determination of the d14 piezoelectric coefficient of tellurium. Solid State Commun. **7**, 161-163 (1969).
10. Jnawali, G., Xiang, Y., Linser, S. M. et al. Ultrafast photoinduced band splitting and carrier dynamics in chiral tellurium nanosheets. Nat. Commun. **11**, 3991 (2020).
11. Agapito, L. A., Kioussis, N., Goddard, W. A. I. et al. Novel family of chiral-based topological insulators: elemental tellurium under strain. Phys. Rev. Lett. **110**, 176401 (2013).
